# Understanding asthma and the metabolic syndrome - a Nigerian report

**DOI:** 10.1186/1755-7682-5-20

**Published:** 2012-06-22

**Authors:** Olufunke O Adeyeye, Anthonia O Ogbera, Olayinka O Ogunleye, Ayodeji T Brodie-Mens, Folasade F Abolarinwa, Raymond T Bamisile, Babatunde O Onadeko

**Affiliations:** 1 Department of Medicine, Lagos State University College of Medicine, 1/5 Oba Akinjobi G.R .A. P.M.B, Ikeja, Lagos, 21266, Nigeria; 2 Department of Pharmacology, Lagos State University College of Medicine, 1/5 Oba Akinjobi G.R .A. P.M.B, Ikeja, Lagos, 21266, Nigeria

## Abstract

**Introduction:**

Nigeria is a developing country that is currently witnessing an upsurge in diabetes mellitus and obesity with its antecedent consequences. There is also a fairly high prevalence of asthma affecting an estimated 10.7% of the population. There is no data presently on the possible presence of metabolic syndrome in Nigerian living with asthma. The study was conceived to determine the prevalence of metabolic syndrome among a population of asthmatics seen in our practice. We also attempt to compare asthma severity, control and pulmonary function tests in asthmatics with metabolic syndrome and those without.

**Methodology:**

This cross-sectional study was carried out at the asthma clinic of a tertiary teaching hospital. Ethical clearance was obtained from the research and ethics committee of the hospital. Written consent was obtained from the participants. Interviewer based questionnaire was used to obtain required information, anthropometric indices were recorded and clinical examinations done. Pulmonary function tests were carried out using desktop Alpha Spirometer model 6000 made by Vitalograph UK (2007). Blood pressure was measured using sphygmomanometer in mmHg. Fasting venous blood was taken for blood sugar and lipid profile. Metabolic syndrome was defined by the international diabetes Federation (IDF) criteria.

**Result:**

One hundred and fifty eight (158) asthmatics participated in the study comprising of 63 (39.9%) males and 95(60.1%) females. The age range was 14-78 years with a mean of 46.48+/-17.00 years. The mean duration of asthma diagnosis was 13.95+/-12.14 years. The prevalence of hypertension was 29.1%. 17 (10.8%) had fasting blood sugar above 100 mg/dl. Abdominal obesity was present in 78 (49.5%). The mean total cholesterol was 192.63+/-40.7 mg/dl. HDL was low in 21(22%) of female and 3 (4.8%) male. The prevalence of metabolic syndrome was 17.7%, affecting 28 asthma patients. Asthma control was affected by the presence of metabolic syndrome. P < 0.05. The pulmonary function test was not significantly affected by presence of metabolic syndrome.

**Conclusion:**

Metabolic syndrome prevalence is high in the population of asthma patients studied. It is therefore important to screen patient with asthma for this condition and treat to improve outcome.

## Background

Asthma affects an estimated 300 million people worldwide [1]. It is a chronic inflammatory disorder associated with airway hyper responsiveness which results in recurrent episodic wheezing, chest tightness and breathlessness. The worldwide prevalence of asthma is estimated to range from 1-18% and in Nigeria about 13% of the populace is affected [2-5]. The basic risk factor for development of asthma remain complex and it is established that genetics and the environments overlap with other factors like obesity and gastroesophageal reflux disease (GERD) to influence asthma [6]. Although asthma and obesity, are two fast growing health problems of public health significance in developing countries, the relationship between these two disorders remains at best shrouded in mystery. It is pertinent to note that the presence of obesity creates unique challenges for the diagnosis and treatment of asthma. Some researchers have noted that there is growing evidence of the influence of hyperglycaemia and hyper-insulinaemia which are key features of the metabolic syndrome on airway function and structure [7]. The increasing prevalence of hypertension and lipid abnormalities as well as diabetes also poses some challenges in addition to the rising health care cost.

Several studies have associated obesity with asthma particularly central obesity which is an important component of metabolic syndrome. There is growing epidemiological evidence that obesity increases the risk of developing asthma. In some studies, insulin resistance or metabolic syndrome is a stronger risk factor than the body mass [8].

The obese-asthma sub phenotype is thought to be characterized by paucity of inflammation but marked symptoms, poor response to glucocorticoids, and peripheral airway dysfunction. While obesity may predispose to increased Th2 inflammation or atopic tendencies, other mechanisms that are independent of inflammatory cells, need to be considered. There is growing evidence of the influence of hyperinsulinemia and insulin-like growth factors on airwaystructure and function [9-11].

The metabolic syndrome is a cluster of risk factors that include hypertension, impaired glucose tolerance or diabetes mellitus, central obesity and dyslipidaemia. Nigeria is a developing country that is currently witnessing an upsurge in diabetes mellitus and obesity and the attendant sequelae. Against a background of a fairly high prevalence rate of asthma, the disease burden of these disorders may ultimately prove to be unacceptably high.

Hitherto, there has been no data on the possible presence e of the metabolic syndrome in Nigerians living with asthma. Thus this study was carried out to determine the prevalence of metabolic syndrome among a population of asthmatics seen in our practice and also compare the asthma severity, control and pulmonary function amongst those with metabolic syndrome and those without the metabolic syndrome.

## Methodology

This was a cross sectional study carried out at the asthma clinic of Lagos State University Teaching Hospital Ikeja. The hospital is one of the three tertiary hospitals in the megacity of Lagos with about 18 million residents. The hospital receives referral from secondary private and public health institutions in the state. The asthma clinic is conducted by Pulmonologists and resident doctors.

Ethical approval was obtained from the hospital research and ethics committee. All participants gave written informed consent prior to enrolment in the study. Written informed consent was obtained from parents of willing patients who were below 18 years. Patients enrolled into the study were those on follow up at the asthma clinic with established diagnosis of bronchial asthma based on the pattern of symptoms (airways obstruction and hyperresponsiveness) and/or response to therapy (reversibility) over time [1].

Asthma patients with an F score of < 8 recorded from a previous study on the prevalence of gastroesophageal reflux disease (GERD) among asthmatics were excluded [12]. An information that was accessed from the clinic registry and previous data base for every patient on follow up. All consenting consecutive patients on follow up who met these criteria were enrolled into the study over a period of six months between June and December 2010.

Interviewer administered questionnaires were used to collect information on the bio data of the patients, the asthma symptoms, duration and asthma control. Anthropometric indices were determined by standard criteria and the blood pressure measured using a mercury sphygmomanometer with the systolic and diastolic blood pressures taken as the first and fifth korotkoff sounds respectively.

The pulmonary function tests were done in the sitting position using desktop Alpha Spirometer model 6000 made by Vitalograph UK (year 2007) to measure the FEV1, FVC, PEFR and post bronchodilator responses following inhalation of 200ug salbutamol.

Laboratory assessments included venous blood samples in a fasted state for the determination of components of the lipid panel (total cholesterol, high density cholesterol, low density cholesterol and triglyceride) and blood glucose levels. The serum glucose was measured using the glucose-oxidase method and the lipid profile by the enzymatic-colorimetric method.

The following operational definitions were utilized:

1. Asthma was diagnosed based on episodic breathlessness, chest pain and wheeze with reversible airway obstruction [1].

2. The presence of three or more of any of the following is a pointer to the metabolic syndrome. Waist circumference (WC) greater than 102 cm in men and 88 cm in women; serum triglycerides (TG) level of at least 150 mg/dL (1.69 mmol/L); high-density lipoprotein cholesterol (HDL-C) level of less than 40 mg/dL (1.04 mmol/L) in men and 50 mg/dL (1.29 mmol/L) in women; blood pressure of at least 130/85 mm Hg; or serum glucose level of at least 100 mg/dL (5.6 mmol/L) [13].

3. Asthma control was defined according to GINA (Global Initiative for Asthma.) Controlled asthma is said to be present if there is none or less than twice a week Daytime symptoms, No limitation of activities, no nocturnal symptoms or awakening, need none or less than twice a week need for reliever/rescue medications and the Lung Functions (Peak Expiratory Flow rate or Forced Expiratory volume in one sec (FEV1) is normal. The asthma is said to be partly controlled if any of the following is present: More than twice per week daytime symptoms, any limitation of activity, nocturnal awakening needing more than twice/week rescue or reliever treatment and PEF or FEV1 less than 80% predicted. The asthma is uncontrolled if three or more of the partly controlled asthma is present [1].

4. Asthma severity was classified into intermittent, mild persistent, moderate persistent and severe persistent using the symptoms and PEFR according to GINA guidelines [1].

5. Cadre of BMI were classified as follows; Anthropometric indices were classified into underweight (<18.5 kg/m^2^), healthy/normal (18.5 to 24.9 kg/m^2^), overweight (25 to 29.9 kg/m^2^), and obesity (30 kg/m^2^) [14,15].

6. Lung function tests refer to the Forced Expiratory Volume in one second (FEV1), forced vital capacity (FVC) as well as the Peak Expiratory flow rate (PEFR).

Data analysis was done using the SPSS version 17. Descriptive statistics was done and quantitative variable expressed as means ± SD. The qualitative data were expressed in ratio or proportions. Comparison of means were done using the student ‘t’ test and anova as applicable while categorical variables were compared with the chi square test. All tests were performed at 5% level of significance.

## Results

### Baseline characteristics of the study subjects

A total of 158 asthma patients were enrolled into the study within the study period having fulfilled the enrolment criteria aforementioned and having given a written informed consent. There were 63 (39.9%) males and 95 (60.1%) females. The age range was between 14 and 78 years with a mean age of 46.5 ± 17.2 years. There was no significant gender difference in mean age of the participants: males were 44.9 ± 16.2 years and 47.6 ± 17.7 years for the females (p = 0.334). The females had a longer duration of asthma than the males with mean duration of 16.1 ± 13.4 years compared with 10.7 ±9.2 years for males, p = 0.006.

Metabolic syndrome was present in 28 patients (17.7%) comprising of 13 males and 15 females. The socio demographic characteristics of asthmatics with metabolic syndrome and those without metabolic syndrome were compared in Table 1. Asthmatics with metabolic syndrome were significantly older than those without while those without metabolic syndrome seemed to have higher educational levels attained than those with metabolic syndrome. No difference was noted in the sex distribution between the two categories.

**Table 1:** Comparison of socio-demographic characteristics and the baseline metabolic profiles of asthmatics with metabolic syndrome and those without metabolic syndrome

**Variables**	**Asthmatics with metabolic syndrome**	**Asthmatics without metabolic syndrome**	**p value**
**Mean age(Years)**	52.64 ± 11.67	45.15 ± 11.67	0.007
**Sex (Frequency/%)**			
**Male**	13.0(46.4)	50.0(38.5)	
**Female**	15.0(53.6)	80.0(61.5)	0.500
**Educational level (Frequency/%)**			
**None**	1.0(3.6)	37.0(27.6)	
**Primary**	24.0(85.7)	73.0(54.5)	
**Secondary**	0.0(0.0)	6.0(4.5)	
**Tertiary**	3.0(10.7)	14.0(10.4)	0.015
**Mean duration of asthma (Years)**	14.6 ± 9.8	13.8 ± 12.6	0.700
**Mean FBS(mg/dl)**	86.2 ± 18.4	82.4 ± 12.0	0.192
**Mean total fasting cholesterol(mg/dl)**	224.6 ± 36.7	185.7 ± 38.3	<0.001
**Mean triglyceride(mg/dl)**	64.6 ± 12.6	62.0 ± 13.2	<0.001
**Mean HDL-Chol(mg/dl)**	58.5 ± 9.5	51.7 ± 7.5	<0.001
**Mean LDL-Chol(mg/dl)**	143.9 ± 35.7	117.6 ± 30.9	<0.001

HDL-Chol = High density lipoprotein-Cholesterol. LDL-Chol = Low density lipoprotein -Cholesterol.

### Asthma and the metabolic syndrome

**Figure 1:**
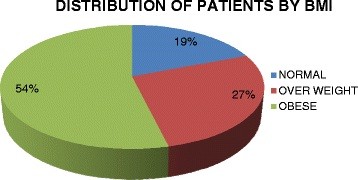
Distribution of studied asthma patients by Body Mass Index (BM).

**Figure 2:**
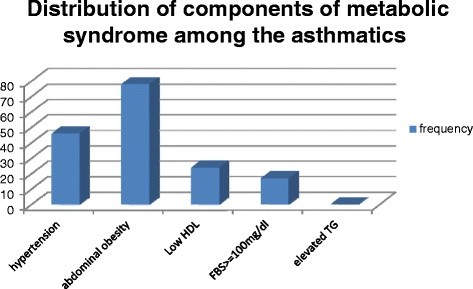
Distribution of the components of metabolic syndrome among the asthmatics.

There were 46 (29.1%) asthmatics with hypertension. The prevalence of obesity according to BMI was 53.8% with 85 patients being obese. The distribution of patients according to BMI is shown in Figure 1. None of the patient was underweight. Majority of the asthmatics had fasting blood sugar less than 100 mg/dl. Only 17(10.8%) had FBS ≥100 mg/dl. Previous diagnosis of diabetes was present in 7(4.4%) of the patients. Abdominal obesity was present in 78 patients (49.4%) comprising of 60 (38%) females and 18 (11.4%) males. Figure 2 illustrates the component of metabolic syndrome found in this population of asthma patients with abdominal obesity being the most prevalent and elevated triglyceride the least.

The mean total cholesterol, mean high density lipoprotein cholesterol (HDL), mean triglyceride (TG), low density lipoprotein cholesterol (LDL) and very low density lipoprotein cholesterol (VLDL) of the patients were 192.6 ± 40.7 mg/dl, 52.9 ± 8.3 mg/dl, 62.5 ± 13.1 mg/dl, 122.2 ± 33.21 mg/dl and 13.3 ± 3.2 mg/dl respectively. Low level of HDL was present in 21(22.1%) of the females (HDL less than 50 mg/dl) while only 3 (4.8%) males had low HDL i.e. less than 40 mg/dl . All the patients had normal level of triglycerides.

Table 1 also shows the comparisons of the metabolic profiles of those with and without metabolic syndrome. Apart from the fasting blood sugar which was comparable in the two groups, those with metabolic syndrome had significantly higher fasting total cholesterol, triglyceride, low density lipoprotein-cholesterol and high density lipoprotein-cholesterol fractions.

### Medications in use by asthma patients

Inhaled corticosteroid and Long acting β agonist (LABA + ICS) were frequently used by the patients 126 (79.7) compared with 32 (20.3). The asthmatics without metabolic syndrome used 109 (83.8) used LABA + ICS significantly more compared with those without metabolic syndrome 17 (60.7) p < 0.05 similarly oral β agonist were used more in those with metabolic syndrome. P < 0.05 There was no statistical significant difference in the usage of other medications between the two groups. This is shown in Table 2.

**Table 2:** Medications in use by the asthma patients

**Medications**	**Asthmatics with metabolic syndrome**	**Asthmatics without metabolic syndrome**	**P value**
LABA + ICS	17(60.7)	109(83.8)	0.009
ICS alone	10(35.7)	53(40.8)	0.392
Oral Prednisolone	7(25)	31(23.8)	0.534
β agonist	22(78.6)	103(81.1)	0.469
Oral Theophylline	5(17.9)	13(10.2)	0.203
Oral β agonist	11(39.3)	13(10.2)	0.000
Zarfilukast	3(10.7)	3(2.3)	0.069

LABA + ICS long acting β agonist combined with inhaled corticosteroid.

### Asthma control and severity

Table 3 compared asthma control and severity between those with and without the metabolic syndrome. Those without metabolic syndrome had a better asthma control than those with metabolic syndrome. No difference existed in disease severity between the two groups.

**Table 3:** Comparisons of asthma control and severity in those with metabolic syndrome and those without metabolic syndrome

**Parameters (frequency/%)**	**Asthmatics with metabolic syndrome**	**Asthmatic without metabolic syndrome**	**p value**
**Asthma control**			
Well Controlled	4.0 (2.5)	41.0 (25.9) 0.03	
Partial Control	8.0 (4.4)	49.0 (31.0)	
Uncontrolled	6.0 (3.8)	40.0 (25.3)	
**Asthma severity**			
Intermittent	11.0 (7.0)	52.0 (32.9)	
Mild Persistent	2.0 (1.3)	33.0 (20.9)	
Moderate Persistent	10.0 (6.3)	29.0 (18.4)	
Severe Persistent	5.0 (3.2)	16.0 (10.5)	0.129

### Pulmonary function tests

Table 4 shows the comparisons of the pulmonary function tests between the two groups .It was noted that there was no significant difference in the mean predicted FEV1, FVC, obtained PEFR, obtained FEV1, and obtained FVC of asthma patients with metabolic syndrome and those without metabolic syndrome. The post bronchodilatory responses in the two groups did not show any significant difference as well.

**Table 4:** Comparisons of pulmonary functions in asthmatics with metabolic syndrome and those without metabolic syndrome

**Pulmonary function**	**Asthmatics with metabolic syndrome**	**Asthmatic without metabolic syndrome**	**p value**
**Predicted FEV1/L**	2.26 ± 0.51	2.46 ± 0.65	0.129
**Predicted FVC/L**	2.72 ± 0.62	2.94 ± 0.77	0.161
**Predicted PEFR (L/min)**	393.6 ± 74.0	385.2 ± 72.0	0.596
**Obtained PEFR (L/min)**	247.0 ± 121.0	293.7 ± 141.0	0.110
**Obtained FEV1/L**	1.54 ± 0.78	1.86 ± 0.93	0.960
**Obtained FVC/L**	2.27 ± 0.85	2.64 ± 1.05	0.083
**Post Bronchodilator**	1.71 ± 0.68	2.06 ± 0.90	0.056
**FEV1/L**			
**Post Bronchodilator**	2.56 ± 0.67	2.88 ± 1.00	0.072
**FVC/L**			

FEV1 = Forced Expiratory Volume in 1second,FVC = Forced Vital Capacity, PEFR = Peal Expiratory Flow Rate.

### Co-morbidities in persons with asthma

The prevalence of diabetes was 4.4% with 7 patients having diabetes. Hypertension was present in 46(29.1%) of the patients. Allergic Rhinitis, allergic sinusitis, allergic conjunctivitis, and atopic dermatitis was present in 77(48.7%), 56 (35.4%), 66 (41.8%) and 6 (3.8%) respectively. Majority 114 (72.2) of the patients did not have family history of asthma.

## Discussion

In this cross-sectional study of a subset of asthma patients, we found metabolic syndrome in 17.7% of the asthma patients studied. This is rather high and may be a reflection of the increasing prevalence of the metabolic syndrome in the population. Almost half of the asthmatics studied had abdominal obesity. This is quite worrisome as abdominal obesity remains one of the important components of the metabolic syndrome. In a similar study by Uzunlulu et al. the prevalence of metabolic syndrome in asthma patients was 36.7% which is higher than our own study but when compared with their own control they did not find any statistically significant difference [16]. Obesity and asthma are both characterised by inflammation. Obesity leads to pro-inflammatory milieu characterised by increased levels of TNF-α, IL-6, leptin, angio-tensinogen, plasminogen activation inhibitor-1 and decreased adip-onectin levels which promote endothelial functions. The TNF-α is also involved in the initiation of allergic airway responses in asthma. Meta-analysis has shown association between obesity and development of asthma [8].

In our study we found that the patients with metabolic syndrome co-existing with their asthma were much older than those having asthma without metabolic syndrome. This is not surprising as diseases like hypertension, Type 2 diabetes mellitus (Type 2-DM) and dyslipidaemia tends to occur more in the older age groups. The same cannot be said of obesity, an important component of the metabolic syndrome which is on the increase even among the younger age groups. The prevalence of obesity in this study is unacceptably high. This is not surprising because according to the World Health Organisation (WHO), obesity has reached epidemic proportions globally with more than 1 billion adults being overweight and at least 300 million of them clinically obese. Obesity is now recognised by WHO as a major contributor to the global burden of chronic diseases and disability [14]. It is therefore important for clinicians particularly those practicing in developing countries to be alert and sensitive to early diagnosis of obesity in all patients with chronic conditions including asthma.

The relative increase in the prevalence of abdominal obesity in the females when compared with males is also in keeping with the literature since women have been documented to suffer a disproportionate burden of disease attributable to overweight and obesity. It is believed that female obesity has increased by 15% in the past decade and as a result has become more prone to diseases like diabetes and hypertension. Some of the reasons ascribed to this include low level of physical activity, genetic factors and endocrine factors among others [14].

In this study, hypertension existed in a significant proportion of the asthma patient and this may be a reflection of the prevalence of hypertension in the population or a consequence of steroid therapy in use by the asthmatics. Although in our study we did not compare our findings to the general population or those without asthma, this is one of the limitations of our study. However an extensive study requiring larger number of asthmatics and matched with a control without asthma will be needed in the future to answer some of the questions.

In our study we did not find any significant association between presence of metabolic syndrome and pulmonary function tests. This may be the consequence of the fact that our asthma patients are those previously diagnosed and already on treatment for asthma being followed up by specialist physicians this is at variance with Leone who found an independent relationship between lung function impairment (FeV1 or FVC < lower limit of normal) and metabolic syndrome in both sexes [17].

In our study we could not exclude the potential role of obstructive sleep apnea on respiratory system in our obese asthmatics. This is because none of the obese asthmatics had any sleep studies done and we were unable to evaluate the adipokines and pro-inflammatory cytokines which are often up regulated in obese patients and could impact on the neural control of the upper airway in obstructive sleep apnoea. These are very important limitations in our study.

## Conclusion

The prevalence of metabolic syndrome is high among the asthma patients studied. We also recorded poorer asthma control in those with the metabolic syndrome. It is therefore important to screen patient with asthma for the components of metabolic syndrome and institute appropriate managements when indicated to improve overall outcome of asthma management.

## Competing interests

Olufunke O Adeyeye , Anthonia O Ogbera, Olayinka O Ogunleye, Ayodeji T Brodie-Mens, Folasade F Bolarinwa, Raymond T Bamisile, Babatunde O Onadeko. We have no competing interest.

## Authors’ contributions

OO conceived of the study, and participated in its design and coordination as well as writing of the manuscript. OA involved in the drafting, conception, design, collection and analysis, writing of the manuscript. OO participated in its design data collection and analysis. AT analyse the biochemical parameters. FF carry out pulmonary function test on participants. RT collection of data, manuscript revision. BO involved in the drafting, conception, design and collection. All authors read and approved the final manuscript. The study was funded by the Authors.
